# Classification and characterisation of livestock production systems in northern Tanzania

**DOI:** 10.1371/journal.pone.0229478

**Published:** 2020-12-30

**Authors:** William A. de Glanville, Alicia Davis, Kathryn J. Allan, Joram Buza, John R. Claxton, John A. Crump, Jo E. B. Halliday, Paul C. D. Johnson, Tito J. Kibona, Blandina T. Mmbaga, Emmanuel S. Swai, Christopher B. Uzzell, Jonathan Yoder, Jo Sharp, Sarah Cleaveland

**Affiliations:** 1 Institute of Biodiversity, Animal Health and Comparative Medicine, College of Medical, Veterinary and Life Sciences, University of Glasgow, Glasgow, United Kingdom; 2 School of Social and Political Sciences, University of Glasgow, Glasgow, United Kingdom; 3 Nelson Mandela African Institution of Science and Technology, Arusha, Tanzania; 4 Centre for International Health, University of Otago, Dunedin, New Zealand; 5 Division of Infectious Diseases and International Health, Duke University Medical Center, Durham, NC, United States of America; 6 Duke Global Health Institute, Duke University, Durham, NC, United States of America; 7 Kilimanjaro Christian Medical University College, Tumaini University, Moshi, Tanzania; 8 Kilimanjaro Clinical Research Institute, Moshi, Tanzania; 9 Department of Veterinary Services, Ministry of Livestock and Fisheries, Dhaka, Tanzania; 10 School of Geographical and Earth Sciences, University of Glasgow, Glasgow, United Kingdom; 11 School of Economic Sciences, Washington State University, Pullman, WA, United States of America; 12 School of Geography and Sustainable Development, University of St Andrews, St Andrews, United Kingdom; International Maize and Wheat Improvement center (CIMMYT), MEXICO

## Abstract

Livestock keepers in sub-Saharan Africa face a range of pressures, including climate change, land loss, restrictive policies, and population increase. Widespread adaptation in response can lead to the emergence of new, non-traditional typologies of livestock production. We sought to characterise livestock production systems in two administrative regions in northern Tanzania, an area undergoing rapid social, economic, and environmental change. Questionnaire and spatial data were collected from 404 livestock-keeping households in 21 villages in Arusha and Manyara Regions in 2016. Multiple factor analysis and hierarchical cluster analysis were used to classify households into livestock production systems based on household-level characteristics. Adversity-based indicators of vulnerability, including reports of hunger, illness, and livestock, land and crop losses were compared between production systems. Three distinct clusters emerged through this process. The ethnic, environmental and livestock management characteristics of households in each cluster broadly mapped onto traditional definitions of ‘pastoral’, ‘agro-pastoral’ and ‘smallholder’ livestock production in the study area, suggesting that this quantitative classification system is complementary to more qualitative classification methods. Our approach allowed us to demonstrate a diversity in typologies of livestock production at small spatial scales, with almost half of study villages comprising more than one production system. We also found indicators of change within livestock production systems, most notably the adoption of crop agriculture in the majority of pastoral households. System-level heterogeneities in vulnerability were evident, with agro-pastoral households most likely to report hunger and pastoral households most likely to report illness in people and livestock, and livestock losses. We demonstrate that livestock production systems can provide context for assessing household vulnerability in northern Tanzania. Policy initiatives to improve household and community well-being should recognise the continuing diversity of traditional livestock production systems in northern Tanzania, including the diversity that can exist at small spatial scales.

## Introduction

Livestock play a key role in the livelihoods of many households in low-income countries where they contribute to informal household insurance and financing, soil fertility, and household nutrition [1]. In Tanzania, 50% of all households keep livestock, with the sale of products derived from animals constituting an average of 15% of the annual income of rural livestock-keeping households [2]. Livestock provide the social, cultural, and economic backbone to many rural communities in low-income settings, particularly those in marginal, semi-arid and arid environments. Here, the mobility of cattle, sheep, goats, and camels allows livestock keepers to utilise grazing and browsing on common land over a potentially wide geographic area [3], optimising production and reducing household vulnerability to the effects of local rainfall deficits [4]. In these environments, livestock can also provide the security to pursue potentially riskier livelihood activities relying on local rainfall, such as crop agriculture [4]. Supporting livestock production among the rural poor can provide an important route toward sustainable development, equitable livelihoods, and household health and welfare [5].

Livestock-based livelihoods are under growing pressure in many low-income countries from a range of sources [6]. These include the effects of climate change which, in East Africa, are expected to include increasing variability in precipitation [7–9]. Such effects are already becoming apparent in the region. In grassland areas of northern Tanzania, for example, the growing season during the ‘long rains’ period has declined from an average of 100 days in 1960 to 63 days in 2010 [10]. Droughts in East Africa are also becoming more frequent and severe. In 2009, during one of the most severe droughts in living memory, up to 90% of livestock in some parts of northern Tanzania died [11]. Changing systems of land tenure, including the conversion of previously communal land to private ownership or wildlife conservation, further contribute to reduced availability of grazing land [12–16]. Livestock keepers in East Africa are therefore having to adapt to rapidly changing circumstances. Examples of adaptation include the adoption of non-traditional livestock species [17,18], new ways of rearing livestock [19], and the diversification of livelihood profiles in semi-arid areas away from livestock-focused production toward mixed livestock and crop agriculture [20,21]. The extent of these changes and their implications for the characteristics and distribution of ‘traditional’ systems of livestock production in countries undergoing rapid social, economic, and environmental change warrants continued examination.

In northern Tanzania, three traditional typologies of livestock production (or livestock production systems) have existed for several centuries [22,23]. These systems of production can broadly be described as ‘pastoral’, ‘smallholder’, and ‘agro-pastoral’. While there has been substantial geographic and social overlap between systems, and their boundaries often hard to define [22], each has traditionally been linked to particular environmental conditions and ethnic groups. Pastoral systems have been found in the semi-arid, rangeland areas of northern Tanzania and historically dominated by Maasai ethnicities, with less populous groups such as the Barabaig also present. This production system has traditionally relied primarily, but not exclusively [22], on livestock production, utilising long distance movements for grazing in response to variable rainfall patterns. Smallholder farming systems, by contrast, have traditionally been found on the high soil fertility slopes of Mount Kilimanjaro, Mount Meru and the Pare mountains. Here, members of ethnicities such as the Chagga, Meru, and Pare have reared typically small numbers of livestock integrated closely with intensive cash and subsistence crop production [23–25]. Agro-pastoral systems in northern Tanzania have also traditionally involved mixed crop and livestock agriculture but have typically been found in more marginal areas. While crop production has generally made the largest overall contribution to agro-pastoral livelihoods [26], large herd sizes with varying levels of mobility have allowed these farmers to maximise the productivity of available grassland [4,27]. Agro-pastoral production in the region has historically been practiced by groups such as the Arusha and Iraqw, with the former having maintained particularly close social, cultural, and economic relationships with pastoral communities [23,28].

In light of livestock keeper adaptation to changing conditions in northern Tanzania, it is uncertain the extent to which these three broad typologies still characterise livestock production systems in the area. Myriad new livestock production typologies could emerge from demographic, technological, and environmental change. For example, a relatively small number of livestock keepers in Tanzania have adopted exclusively commercial production to meet growing demand for livestock products, particularly among urban populations. This has included beef ranching and the establishment of zero-grazing dairy units with European breeds of cattle [2]. The commercialisation and intensification of livestock production is strongly promoted by the Government of Tanzania [29]. Non-traditional production systems that have a greater focus on narrowly defined production objectives rather than subsistence or the socio-cultural utility of livestock are therefore likely to continue to emerge.

New technologies such as mobile telephones [30], new strategies and tools for household health management [31], and changes in land tenure and land availability [32] may also lead to change within traditionally defined production systems. While such adaptive change may increase overall diversity within a particular geographic area, it could also lead to further blurring of the boundaries between production systems. For example, the adoption of crop agriculture by Maasai pastoralists has been reported as a response to changing land tenure practices in northern Tanzania [20,21]. Widespread adoption and subsequent change within this traditional pastoral system could therefore conceivably lead to it becoming broadly indistinguishable (in terms of production) from neighbouring agro-pastoral systems.

An evaluation of current characteristics of livestock production, and the classification of the production systems that exist in northern Tanzania, can contribute to the design of system-specific programmes that can support a range of livestock-based livelihoods [6,33]. It can also provide the basis for monitoring further change in these systems [6,33,34] and a contextual basis for assessing household capacity to anticipate, resist, cope with and recover from the impact of current and future hazards, including disease, climate, political, and economic shocks. Here, we use data generated from a cross-sectional survey of livestock-keeping households in northern Tanzania to classify and characterise livestock production systems in the area. Our main aim was to determine whether the three traditional typologies of livestock production (i.e., pastoral, agro-pastoral, smallholder) persist in northern Tanzania, or whether new systems of production can be identified in the data. We describe the main characteristics of the livestock production systems currently present in northern Tanzania with a particular focus on how the vulnerability of livestock-keeping households varies between production systems.

## Methods

### Study area

This work was conducted as part of the ‘Social, Environmental and Economic Drivers of Zoonotic Disease’ (SEEDZ) project, a large cross-sectional study that focused on human and animal zoonotic disease risk in six contiguous districts in Arusha Region (Arusha, Karatu, Longido, Meru, Monduli, and Ngorongoro Districts) and four contiguous districts in neighbouring Manyara Region (Babati Rural, Babati Urban, Mbulu, and Simanjiro Districts). Arusha and Manyara Regions are home to approximately 16% of all cattle and 26% of all sheep and goats in Tanzania [35,36]. The total human population was 3,119,441 in the 2012 Tanzanian National Census (Tanzanian National Bureau of Statistics, NBS) in an area of 66,461 km^2^. These two regions account for 67% of the total area of the Northern Zone of Tanzania. The study area is made up of a mixture of semi-arid and sub-tropical agro-ecological zones [33].

### Village selection

Households were the unit of interest, with a multistage sampling design used to select households within villages. Villages were selected using a generalised random tessellation stratified sampling (GRTS) approach, which provides a spatially balanced, probability-based sample [37]. The GRTS was performed using the *spsurvey* package [38] in the R statistical environment, version 3.1.1. (http://cran.r-project.org/). Village selection was made from a list of villages compiled from the 2012 National Census. Villages in wards, an administrative unit comprising an average of three villages, classified as ‘urban’ rather than ‘rural’ or ‘mixed’ (i.e., urban and rural) according to the 2012 census were excluded from the selection procedure. Villages inside the Ngorongoro Conservation Area (NCA), a wildlife area in which people and their livestock are permitted to live but in which crop agriculture is prohibited, were also excluded. With these exclusions, there were a total of 553 villages from which selection was made. To ensure sampling across a range of agro-ecological settings, villages in the study area were classified as those in which livestock-rearing, rather than crop agriculture, was considered to be the primary livelihood activity (‘pastoral’ villages) and those in which a mix of crop and livestock were considered as important (‘mixed’ villages). Village classification was performed in consultation with district-level government officials, typically the District Veterinary Officer or District Livestock Officer. Village selection was then stratified based on agro-ecological classifications, with 11 villages selected from those defined as ‘pastoral’ and nine villages from those defined as ‘mixed.’ An additional village in a mixed setting was also selected non-randomly near our field headquarters on the outskirts of the city of Arusha for field trialling. No substantial changes were made to data collection tools after trialling, and we therefore included data collected from households in this village in this analysis.

**Fig 1:**
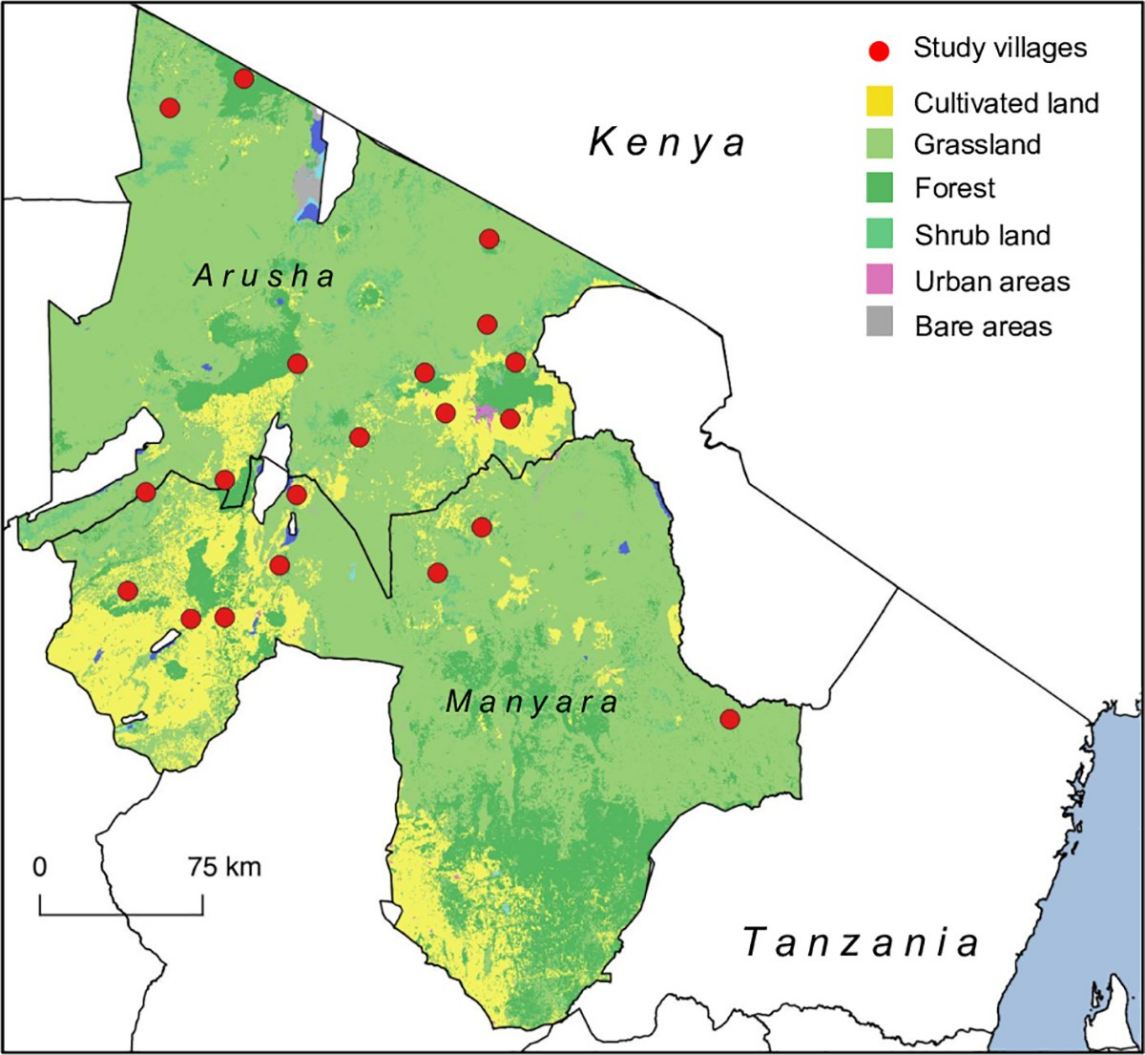
Map of study area in northern Tanzania showing location of study villages in relation to main land classifications in Arusha and Manyara Regions (Map created using QGIS version 2.14.3. Shape files from GADM; landcover raster data from Landsat (https://landsat.gsfc.nasa.gov/data/)).

Fig 1 shows the location of study villages in relation to the main landcover types in northern Tanzania.

### Household surveys

Study villages comprised two to four sub-villages from which two or three were randomly selected for inclusion in the study. Within each sub-village, we adopted a central point sampling approach in which livestock keepers were invited to bring their animals to a pre-selected point within the sub-village, typically a livestock crush or dip tank. Data collection took place alongside sub-village level disease control activities, such as tick or worm control, conducted in collaboration with representatives from the Tanzanian Ministry of Livestock and Fisheries. Village authorities were notified of the proposed event at least three days in advance, with advertisement to livestock keepers in each sub-village performed through the existing village administrative network of chairperson and village elders. During the sampling event, a list of all attending households was generated, and a maximum of ten households were selected from this list using a random number generator. We collected blood samples from animals owned by these households to test for infectious disease exposure (described elsewhere [39,40]). A list of all livestock-keeping households within the sub-village not attending the sampling event was generated with the help of the village chairperson and elders.

On a subsequent day, typically within one-week, recruited households attending the sampling event were visited. During this visit, the household head received an in-depth questionnaire administered in either Kiswahili, Maa, or other local language by trained interviewers. The questionnaire covered a wide range of topics, including household demographics, economics, livestock management practices and livestock health. The geographic co-ordinates of the household were captured using a handheld GPS (Garmin eTrex, Garmin Ltd, Olathe, Kansas, USA). Data collection took place between February and December 2016.

### Ethical approval

All participants provided written informed consent. The protocols, questionnaire tools and consent and assent procedures were approved by the ethics review committees of the Kilimanjaro Christian Medical Centre (KCMC/832) and National Institute of Medical Research (NIMR/2028) in Tanzania, and in the UK by the ethics review committee of the College of Medical, Veterinary and Life Sciences at the University of Glasgow (39a/15). Approval for study activities was also provided by the Tanzanian Commission for Science and Technology (COSTECH) and by the Tanzanian Ministry of Livestock and Fisheries, as well as by regional, district, ward and village-level authorities in the study area.

### Classification of livestock production systems

We used a data-driven approach to classify households into livestock production systems, which we define here as groups of households sharing the same or similar production characteristics [41]. Classification proceeded in two stages. First, we performed dimension reduction using multiple factor analysis (MFA) on a set of household characteristics selected to maximise variation between livestock-keeping households in the study area. This set included household characteristics relating directly to livestock production, crop production, and the local environment as well as to household-level demographic, infrastructural, educational, and nutritional characteristics. Since vulnerability can be considered to be a state of potential adversity, the capacity of a household to prevent or cope with potential hazards (i.e. its vulnerability) can be assessed directly using indicators of adversity, such as death, illness, hunger and land loss [42]. We evaluated production system-level differences in household vulnerability through the inclusion of indicators of adversity in the dimension reduction procedure. Second, hierarchical cluster analysis (HCA) was performed on the output from the MFA with households grouped such that the within-group variability in household characteristics was minimized while between-group variability was maximized. The resulting clusters of households were interpreted to represent distinct and distinguishable livestock production system categories present in the study area at the time of the study. Further details on these two stages are given below.

### Dimension reduction by multiple factor analysis (MFA)

Dimension reduction allows the variability among a set of potentially correlated variables to be represented in terms of a smaller, more parsimonious set of uncorrelated variables. Multiple factor analysis provides a dimension reduction approach for a set of variables describing categorical or continuous data that can be grouped in a meaningful way [43]. Eight groups of variables representing the characteristics of livestock keeping households in northern Tanzania were identified for use in the MFA procedure. These groups were selected based on the authors’ knowledge of the local context and the household-level characteristics that would likely show high levels of variation between livestock keepers in different production systems. The variable groupings (or domains) were: 1. Local household environment; 2. Household demographics; 3. Crop agriculture; 4. Numbers of cattle, sheep and goats owned; 5. Other livestock owned; 6. Livestock management practices; 7. Household food consumption practices; and 8. Adversity-based indicators of household vulnerability. The variables comprising each of these domains are shown in Table 1. The MFA was performed in R using the *FactoMineR* package [44]. Data for household characteristics were derived from the household questionnaire (Domains 2 to 8) or from data extracted at the household-level within a geographic information system (QGIS, version 2.14.3) from publicly available environmental datasets (Domain 1). Further details on the source and manipulations of environmental data and the questions asked at the household level are provided in the S1 File. Up to a maximum of 5% missing-ness was present in around 20% of variables. Imputation of missing values was performed using a regularized iterative MFA algorithm in the *missMDA* package [45] in R. Continuous variables (Domains 1 and 4) with obvious right skew were transformed using a natural logarithm. All continuous variables were scaled to have a mean of zero and a standard deviation of one before performing the MFA.

**Table 1:** Domains and contributing variables for the multiple factor analysis to classify livestock-keeping households into production systems in northern Tanzania.

Domain	Variable	Domain	Variable
1. Environment^1^	Average vegetation cover	4. Livestock	Number of cattle
	Distance to main road (km)		Number of goats
	Travel time to market centre (hours)		Number of sheep
	Annual precipitation (mm)	5. Livestock type	Own pigs
	Average annual temperature (°C)		Own donkeys
	Maximum slope (degrees)		Own chickens
	Local cropland cover (%)		Own exotic breed cattle
	Local grassland cover (%)		Own exotic breed small ruminants
	Local forest cover (%)	6. Management	Cattle transhumance
	Local human population density (km^2^)		Small stock transhumance
	Area of village (decimal degrees^2^)		Graze cattle with small stock
	Local cattle population density (km^2^)		Zero graze cattle
	Local sheep population density (km^2^)		Zero graze small stock
	Local goat population density (km^2^)		Tether cattle
	Local chicken population density (km^2^)		Tether small stock
	Local pig population density (km^2^)		Vaccinate against any disease
2. Household	Sex of household head		Sell milk
	Maasai ethnicity (household head)	7. Consumption	Consumed meat in past 3 days
	Arusha ethnicity (household head)		Consumed dairy
	Meru ethnicity (household head)		Consumed blood
	Iraqw ethnicity (household head)		Consumed vegetables
	Barabaig ethnicity (household head)		Consumed legumes
	Nyaturu ethnicity (household head)		Consumed fat
	Household head completed primary school		Consumed fish
	Government title for land		Consumed poultry
	Has latrine		Consumed root vegetables
	Treat drinking water (including boiling)		Consumed eggs
3. Crops	Growing crops > 10 years		Consumed any animal source food
	Grow no crops	8. Vulnerability	Hunger in past 12 months
	Grow beans		Illness in past 12 months
	Grow cowpeas		Illness in livestock in past 12 months
	Grow maize		Crop losses in past 12 months
	Grow millet		Livestock losses in past 12 months
	Grow onions		Land losses in past 12 months
	Grow potato		
	Grow sesame		
	Grow sorghum		
	Grow sunflower		
	Grow wheat		
	Supplies of staple crops last 6 months or more		
	Own plough		
	Sell crops		

^1^ Further detail on spatial datasets used is given in S1 Table in the S1 File.

### Hierarchical cluster analysis (HCA)

Households were classified into clusters using HCA on the factors (i.e. the set of uncorrelated variables) derived from the MFA. To select which factors to include in the HCA, eigenvalues associated with each factor (describing how much variance is explained) were identified as ‘large’ or ‘small’ based on the presence of a natural break when consecutive eigenvalues were plotted on a scree plot [41]. All factors associated with ‘large’ eigenvalues were included in the clustering procedure. Hierarchical cluster analysis was also performed using the *FactoMineR* package. Ward’s minimum variance criteria were used to derive clusters using the automated procedure within the *FactoMineR* package, with no specification of the number of clusters made *a priori*. The average value of each household characteristic in each of the resulting clusters was compared to the global mean for that characteristic using the v-test. A v-test value greater than 1.96 provides statistical support (i.e. p-value <0.05) for a difference in the mean of the variable in the cluster when compared to the population mean [46].

## Results

Household survey data were collected from 404 households. The median (range) number of households interviewed per village was 19 (7, 30). The average and median proportion of livestock-keeping households in a village attending the sampling event was 45%, with a range between 6 and 100%. The average (median) percentage attending in villages classified as “pastoral” by local experts was 47% (52%) with a range between 10 and 100%. The average (median) percentage attending in villages classified as “mixed” was 43% (36%), with a range between 6 and 100%. Summary statistics for the household characteristics in each domain for all recruited households are given in Tables 2 and 3.

**Table 2:** Mean values for continuous variables for households within clusters derived from hierarchical cluster analysis performed on livestock-keeping households in northern Tanzania (median values are given in square brackets).

		Mean [Median]			
Domains	Variable	Overall (n = 404)	Cluster 1 (n = 171)	Cluster 2 (n = 177)	Cluster 3 (n = 56)
Location	Average annual vegetation cover	0.26 [0.27]	0.23* [0.23]	0.29* [0.29]	0.27* [0.28]
	Distance to main road (km)	36.2 [32.6]	47.8* [10.3]	24.8 [9.4]	7.3* [8.4]
	Time to travel to market centre (hours)	5.4 [3.6]	6.4* [4.6]	5.3 [3.4]	2.6* [1.2]
	Total annual precipitation (mm)	830.4 [818]	742.2* [741]	865.6* [831]	989.9* [912]
	Average annual temperature (°C)	19.3 [19.3]	20.2* [20.0]	18.6* [18.0]	18.6* [18.6]
	Maximum slope (degrees)	3.7 [2.8]	2.2* [1.5]	4.5* [3.3]	4.4* [4.0]
	Local crop land cover (%)	37.6 [25.7]	9.5* [0.00]	51.8* [49.7]	78.3* [92.2]
	Local grassland cover (%)	46.1 [43.1]	73.3* [89.5]	31.1* [24.8]	10.5* [1.2]
	Local forest cover (%)	11.2 [1.3]	9.0 [0.14]	14.3* [5.4]	8.0 [0.58]
	Local human population density (km^2^)	1.4 [0.70]	0.32* [0.17]	1.3* [1.1]	4.7* [1.1]
	Area of village (decimal degrees^2^)	1.8 [0.01]	0.03* [0.03]	0.01* [0.00]	0.00* [0.00]
	Local cattle density (km^2^)	125.7 [0.90]	2.4* [0.45]	39.5* [9.7]	784.3* [80.8]
	Local sheep density (km^2^)	95.4 [4.6]	3.2* [2.7]	10.3* [10.4]	654.7 *[17.1]
	Local goat density (km^2^)	61.6 [1.7]	2.6* [1.27]	17.5* [5.8]	385.4* [41.7]
	Local chicken density (km^2^)	159.9 [9.4]	4.9* [3.04]	42.7* [13.3]	1015.0* [155.0]
	Local pig density (km^2^)	2.0 [0.1]	0.10* [0.02]	1.1* [0.21]	11.2* [0.09]
Livestock	Number cattle	49.6 [10.0]	104.5* [50.0]	10.3* [8.0]	6.5* [5.5]
	Number goats	52.6 [15.0]	107.9* [50.0]	13.6* [8.0]	6.4* [4.5]
	Number sheep	50.0 [10.0]	105.5* [42.0]	8.4* [4.0]	8.8* [3.5]

* v-test value > 1.96 representing statistically significant (p-value <0.05) difference between cluster mean and overall mean.

**Table 3:** Percentages of households reporting variable presence in clusters derived from hierarchical cluster analysis performed on livestock-keeping households in northern Tanzania.

		Percentage of households reporting variable presence			
Category	Variable	Overall^1^ (n = 404)	Cluster 1 (n = 171)	Cluster 2 (n = 177)	Cluster 3 (n = 56)
Household	Household head male	92.3 (89.2–94.6)	94.7	92.1	85.7
	Maasai ethnicity	41.1 (36.2–46.1)	88.9*	5.1*	8.9*
	Arusha ethnicity	20.3 (16.5–24.6)	7.0*	37.9*	5.4*
	WaMeru ethnicity	7.9 (5.6–11.1)	0.0*	1.1*	53.6*
	Iraqw ethnicity	23.3 (19.3–27.8)	0.0*	48.0*	14.3
	Barabaig ethnicity	2.0 (0.9–4.0)	2.9	1.7	0
	Nyaturu ethnicity	2.2 (1.1–4.3)	0.0*	1.1	12.5*
	Head complete primary school	49.3 (44.3–54.2)	26.9*	59.9*	83.9*
	Government title for land	4.0 (2.4–6.5)	3.5	0.0*	16.1*
	Latrine ownership	67.6 (62.7–72.1)	35.1*	89.8*	96.4*
	Treat drinking water	28.0 (23.7–32.7)	26.3	29.9	26.8
Crops	Growing crops > 10 years	71.8 (67.1–76.1)	48.0*	89.8*	87.5*
	Grow no crops	12.9 (9.8–16.6)	26.9*	1.7*	5.4
	Grow beans	60.6 (55.7–65.4)	49.1*	65.5	80.4*
	Grow cowpeas	5.9 (3.9–8.8)	1.8*	10.7*	3.6
	Grow maize	78.7 (74.3–82.5)	58.5*	94.4*	91.1*
	Grow millet	7.7 (5.4–10.8)	1.8*	14.7*	3.6
	Grow onions	5.9 (3.9–8.8)	14.0*	0.0*	0.0*
	Grow potato	4.7 (2.9–7.4)	0.6*	6.7	10.7*
	Grow sesame	3.2 (1.8–5.6)	0.6*	6.2*	1.8
	Grow sorghum	3.0 (1.6–5.3)	0.6*	6.2*	0.0*
	Grow sunflower	9.4 (6.8–12.8)	1.8*	14.1*	17.9*
	Grow wheat	3.0 (1.6–5.3)	0.0*	6.8*	0.0
	Staple crops last ≥ 6 months	59.6 (54.6–64.4)	42.0*	73.3*	69.6
	Own plough	41.8 (37.0–46.8)	28.1*	48.0*	64.3*
	Sell crops	36.6 (31.9–41.6)	29.8*	39.5	48.2
Livestock Type	Pigs	12.6 (9.6–16.4)	0.0*	28.2*	1.8*
	Donkeys	57.7 (52.7–62.5)	86.0*	41.2*	23.2*
	Chickens	85.9 (82.0–89.1)	76.6*	93.2*	91.1
	European breed cattle	2.7 (1.4–5.0)	2.9	0.0*	10.7*
	European breed small stock	2.2 (1.1–4.3)	2.4	0.0*	8.9*
Management	Cattle transhumance	37.8 (32.9–42.8)	76.7*	13.1*	0.0*
	Small stock transhumance	27.3 (22.9–32.1)	59.7*	5.6*	0.0*
	Graze cattle with small stock	25.0 (20.9–29.6)	5.8*	42.3*	28.6
	Zero graze cattle	10.2 (7.5–13.6)	0.0*	0.1*	71.4*
	Zero graze small stock	9.2 (6.6–12.5)	0.0*	0.6*	64.3*
	Tether cattle	4.7 (2.9–7.4)	0.0*	4.0	21.4*
	Tether small stock	5.0 (3.1–7.7)	0.0*	5.1	19.6*
	Vaccinate against any disease	23.4 (19.4–28.0)	38.0*	11.3*	16.3
	Sell milk	15.1 (11.8–19.1)	16.4	7.3*	35.7*
Consumption	Meat	54.2 (49.2–59.1)	75.3*	35.0*	58.9
	Dairy	71.5 (66.8–75.8)	73.7	72.3	62.5
	Blood	6.7 (4.5–9.7)	11.7*	2.3*	5.4
	Vegetables or fruits	69.8 (65.0–74.2)	48.0*	83.6*	92.9*
	Legumes	66.3 (61.4–70.9)	60.2*	70.1	73.2
	Fats	55.0 (50.0–59.0)	55.0	56.5	50.0
	Fish	11.1 (8.3–14.7)	2.3*	10.2	41.1*
	Poultry	15.1 (11.8–19.1)	9.9*	16.4	26.8*
	Root vegetables	28.7 (24.2–33.4)	19.9*	31.6	46.4*
	Eggs	21.5 (17.7–25.9)	12.3*	25.4	37.5*
	Any animal source food	89.4 (85.8–92.1)	91.8	85.9*	92.9
Vulnerability	Hunger	45.0 (40.1–50.0)	48.5	50.8*	16.1*
	Illness in people	60.9 (55.9–65.6)	74.3*	61.0	19.6*
	Illness in livestock	54.7 (49.7–59.6)	67.8*	53.7	17.9*
	Crop losses	34.9 (30.3–39.8)	34.5	40.1	19.6*
	Livestock losses	41.6 (36.8–46.6)	58.5*	36.7	5.4*
	Land losses	27.5 (23.2–32.1)	31.0	31.1	5.4*

^1^ 95% confidence interval given in parentheses

*v-test value > 1.96 representing statistically significant (p-value <0.05) difference between cluster mean and overall mean.

### Multiple factor analysis

**Fig 2:**
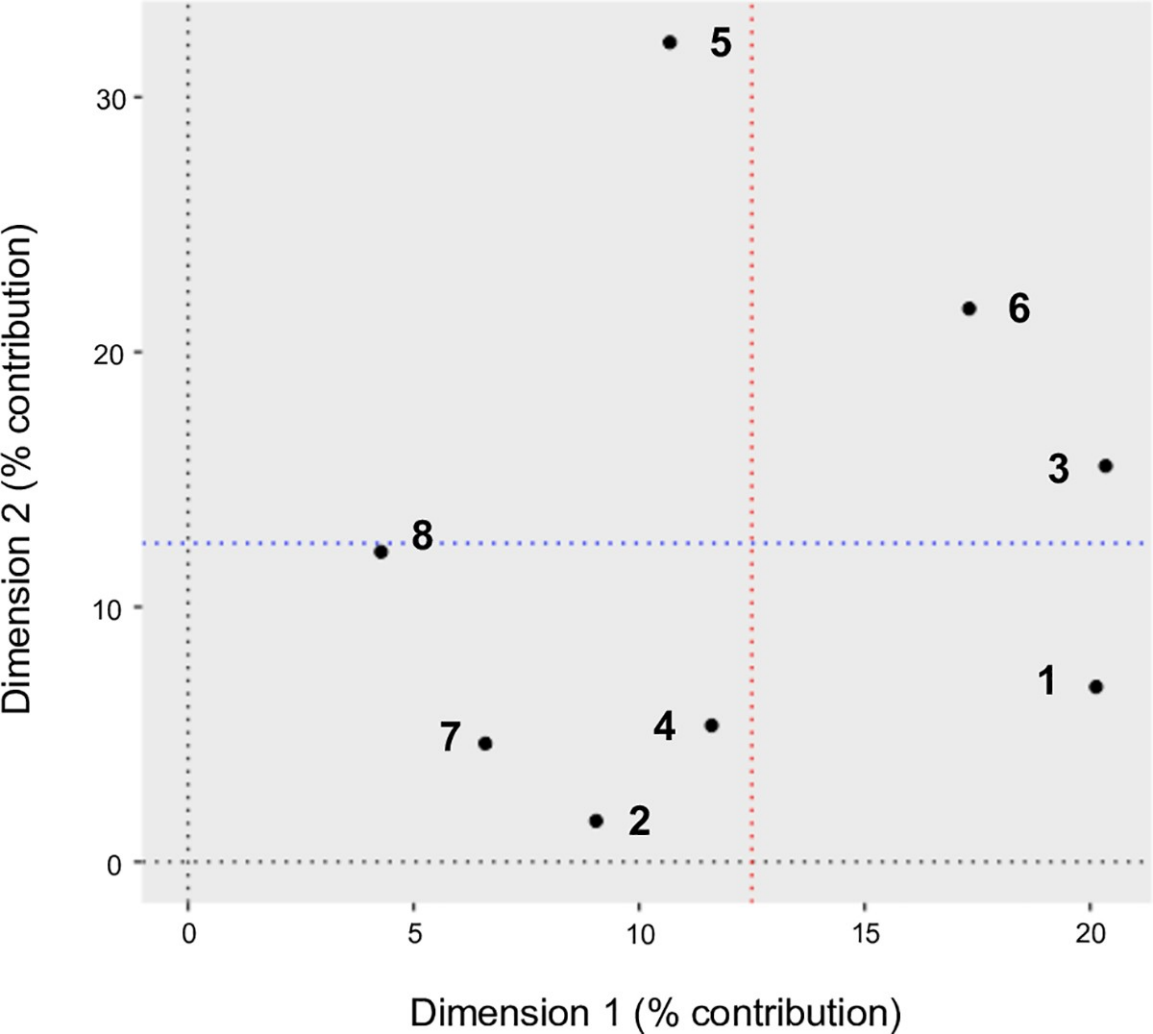
Percent contribution of each group to the first (Dimension 1) and second (Dimension 2) factors derived from MFA performed on characteristics of livestock-keeping households in northern Tanzania. Red (blue) dotted line represents the expected score if all domains contributed equally to the inertia on the first (second) factor (i.e. 100/8 = 12.5%).

The percentage contribution of each domain to explaining variation between households for the first two factors derived from the MFA is shown in Fig 2. The first factor (Dimension 1) explained 14.1% of the total variation, the second factor (Dimension 2) explained 6.3%, with all remaining factors each explaining less than 5%. The percent contribution to the inertia of the first factor was highest for Groups 1 (environment), 3 (crops), and 6 (livestock management) (Fig 2), reflecting the relative importance of these domains in explaining between-household variation.

**Fig 3:**
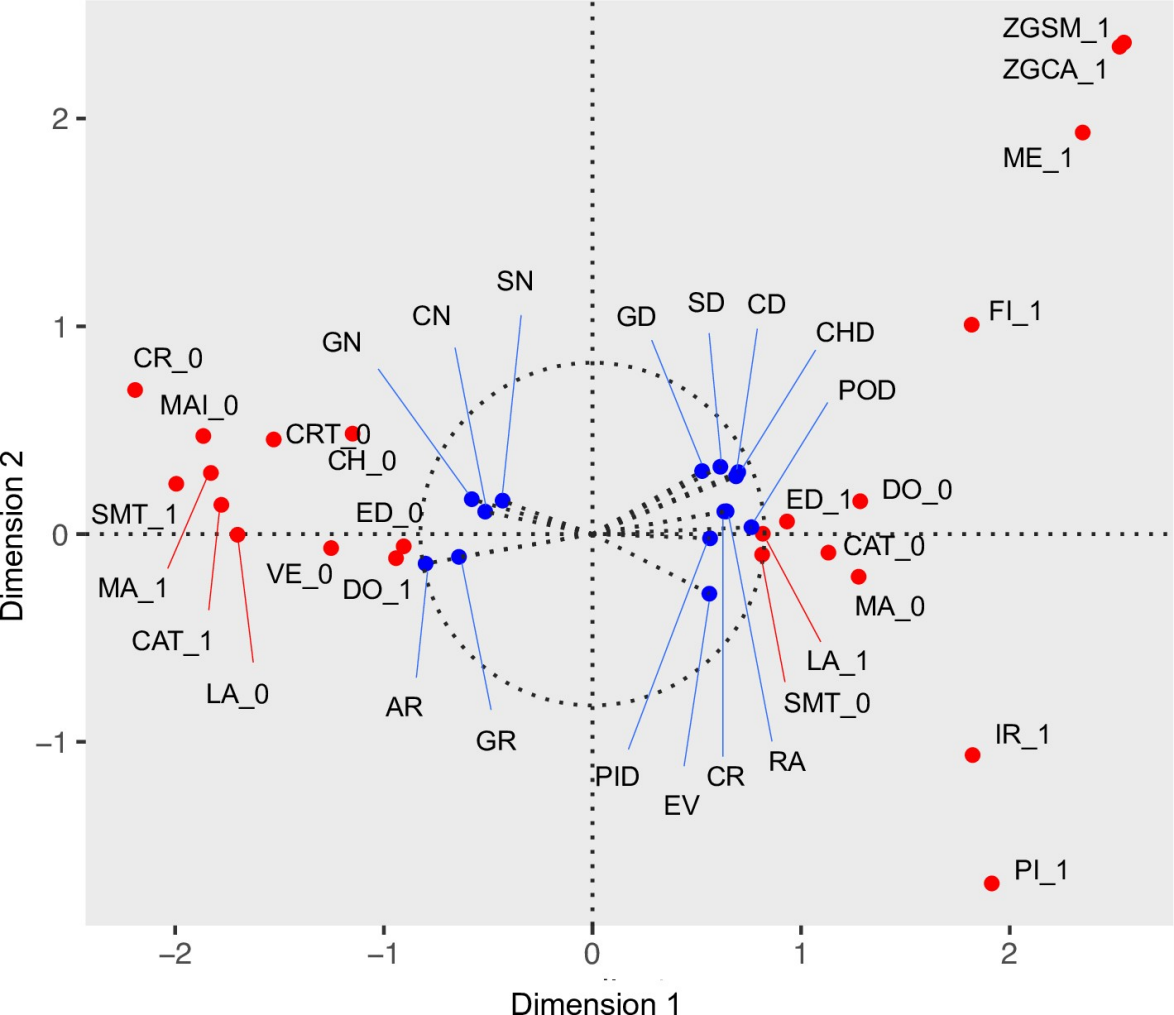
Variable scores in relation to the first and second factors derived from MFA performed on characteristics of livestock keeping households in northern Tanzania. Scores given to categorical (continuous) variables are shown in red (blue). ***Categorical*** (1 indicates presence of described characteristic; 0 indicates absence)**: CAT** = Keep cattle; **CH** = Keep chickens; **CR** = Household grows crops; **CRT** = Grow crops for > 10 years; **DO** = Keep donkeys; **ED** = Household education to primary school or above; **FI** = Household consumed fish in past 3 days; **IR** = Iraqw ethnicity; **LA** = Latrine in household; **ME** = Meru ethnicity; **MA** = Maasai ethnicity; **MAI** = Grow maize; **PI** = Keep pigs; **SMT** = Small ruminant transhumance; **VE** = Household consumed vegetables in past 3 days; **ZGCA** = Zero graze cattle; **ZGSM** = Zero graze small ruminants. ***Continuous***: **AR** = Village area; **CD** = Cattle density; **CHD** = Chicken density; **CN** = Household cattle number; **CR** = Local cropland % cover; **EV** = Enhanced vegetation index; **GD** = Goat density; **GN** = Household goat number; **GR** = Local grassland % cover; **PID** = Pig density; **POD** = Human population density; **RA** = Annual precipitation; **SD** = Sheep density; **SN** = Household sheep number.

Fig 3 shows the scores of those variables that made a contribution to the inertia of the first factor of greater than 1%. The average (median) contribution for included variables was 0.6% (0.2). The four categorical variables making the greatest overall contribution to the first factor were Maasai ethnicity of the household head (4.5%), not keeping donkeys (3.8%), engaging in cattle transhumance (3.4%), and engaging in small ruminant transhumance (3.2%). For the continuous characteristics, the top four variables were number of goats owned by a household (3.9%), number of cattle (3.0%), geographic area of village (2.5%), and local human population density (2.5%). A full breakdown of all variable scores and their contributions to the first and second factors is given in S2 and S3 Tables in the S1 File.

Some clustering in scores of the categorical variables derived from the MFA is visually apparent in Fig 3. This includes the grouping of scores for variables such as Maasai-headed households, households that do not grow crops, or which have not been growing crops for more than 10 years, households engaging in cattle and small ruminant transhumance, households keeping donkeys but not chickens, and households without a latrine or in which the head does not have primary education clustering around negative values for Factor 1 (Dimension 1) and low negative and positive values on Factor 2 (Dimension 2). Scores for variables such as not engaging in transhumance, owning a latrine, some formal education of the household head, not owning a donkey, and grazing cattle with small ruminants cluster around positive values for Factor 1 and low negative and positive values for Factor 2. There was a smaller cluster of scores for Iraqw-headed and pig-keeping households around positive values on Factor 1 and negative values on Factor 2, and a cluster of scores for Meru-headed and zero grazing households around positive values for Factor 1 and 2.

### Hierarchical cluster analysis

**Fig 4:**
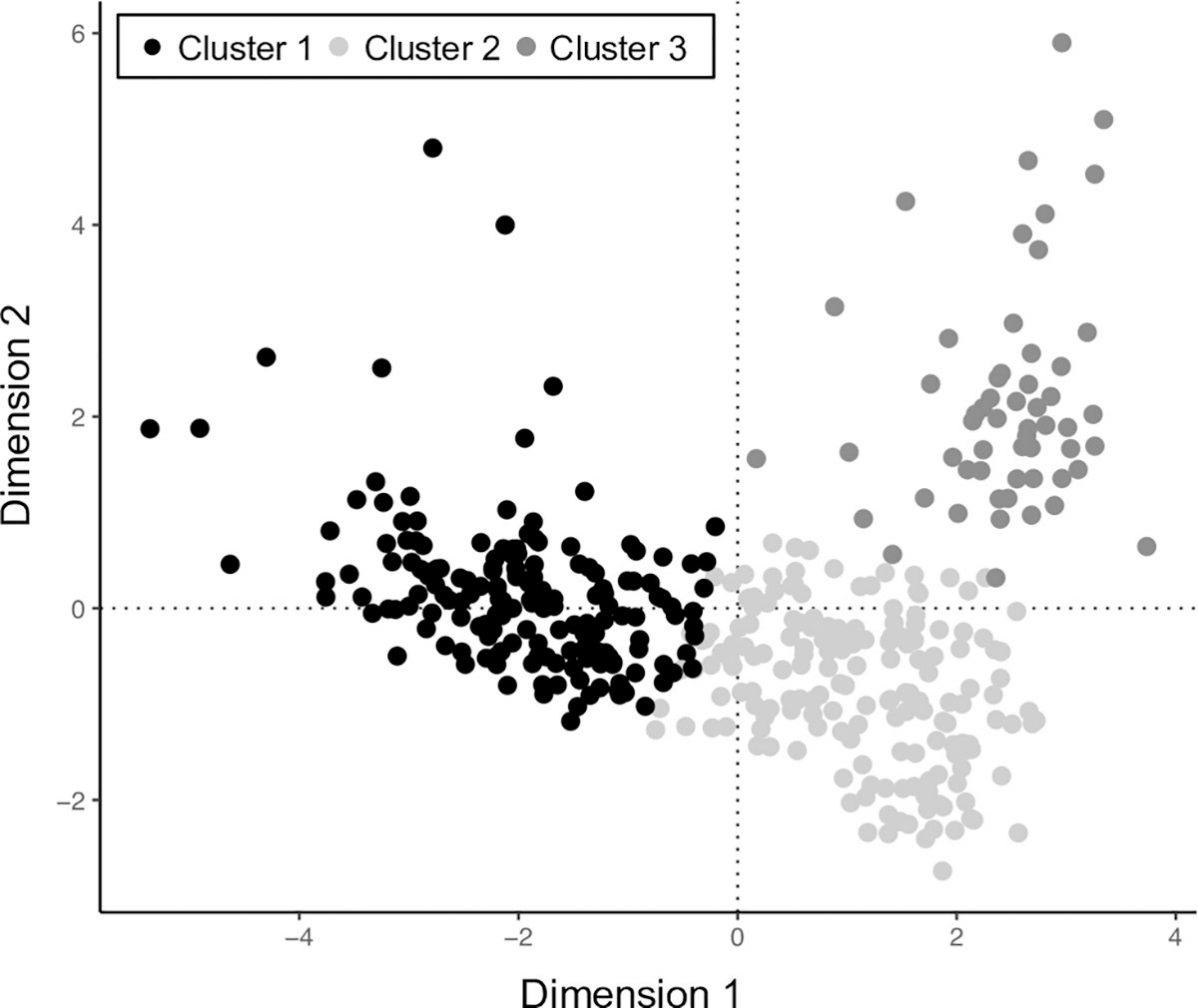
Position of households on the first and second factors (Dimension 1 and 2) derived from the MFA performed on characteristics of livestock keeping households in northern Tanzania. Households are shaded based on cluster membership.

The HCA procedure resulted in the identification of three distinct clusters. The overall score on Factor 1 and 2 for study households and their membership of each cluster is shown in Fig 4. On the basis of the scree plot, the first five factors were included in the clustering procedure (see S1 Fig in S1 File). The composition of each cluster in terms of continuous characteristics is described in Table 2 and in terms of categorical characteristics in Table 3. The majority of continuous and categorical variables in each cluster had a v-test score greater than 1.96, indicating significant differences in the cluster mean when compared to the population mean (Table 3).

The major differences in household characteristics between clusters can be summarised as:

#### Cluster 1

Households in this cluster were characterised as being in areas with low average vegetation cover, having low levels of annual rainfall, low maximum slope (i.e., being relatively flat), low proportion of crop cover, and low population densities of both people and livestock. Cluster 1 households tended to be far from a main road and to have high average travel time to a market centre. Average annual temperature, village area, and proportion of local grassland cover were higher than average for households in the study area. Households in this cluster also had large average herd sizes for cattle, sheep, and goats, and were typically headed by individuals with Maasai ethnicity, with 152 (91.6%) of 166 Maasai-headed households being found in this cluster. Other ethnicities found in this cluster included 12 (14.6%) of all 82 Arusha-headed households, 5 (63%) of the 8 Barabaig-headed households, and 1 (50%) of 2 Datoga-headed households. The majority of household heads in this cluster were without formal education beyond primary school and the proportion of households with a latrine was substantially lower than in the other two clusters. The majority of households reported growing crops in the past year, although this proportion was lower than in the other two clusters. Households growing onions were only found in this cluster. A relatively small proportion of households reported growing millet, sesame, or sunflower. A range of livestock management practices were commonly reported in this cluster, with households more commonly reporting transhumance for both cattle and small ruminants and using livestock vaccination in the past 12 months than households in the other two clusters. No households in this cluster reported zero grazing or using tethered grazing for cattle or small ruminants. No households in this cluster reported keeping pigs, but they commonly kept donkeys. Consuming meat in the past three days was more commonly reported in this cluster than average.

#### Cluster 2

Households in this cluster typically had heads of Arusha and Iraqw ethnicity, including 67 (81.7%) out of 82 and 85 (90.4%) out of 94 of all households with heads with those ethnicities, respectively. Other ethnicities making up this cluster included 9 (5.4%) of the 166 Maasai-headed households, 3 (38%) out of 8 Barabaig-headed households, 1 out of 2 Datoga-headed households, 2 (6.3%) out of 32 Meru-headed households, 1 out of 2 Nyiramba-headed households and 2 (22.2%) out of 9 Nyaturu-headed households. All of the Burunge- (1), Luguru- (1), Rangi- (1), Sandawe- (3), and Sukuma- (1) headed households were in this cluster. The mean, median and percentage values of most contributing variables in this cluster of households tended to fall between those for Clusters 1 and 3, with some exceptions. This cluster of households tended to be in areas with higher average vegetation cover and higher proportion of local forest cover than the average for the study area. No households in this cluster reported having a government title for their land. Most households in this cluster reported growing crops in the past 12 months, with households growing cowpeas, millet, sesame, sorghum, and wheat most likely to be found in this cluster, as were households owning pigs and co-grazing cattle with small stock. They were least likely to report consuming meat over the past 3 days. Levels of livestock vaccination against any disease were lowest in this cluster. Households in this cluster were found in areas with the highest median pig population density. This was the largest cluster (Table 2).

#### Cluster 3

Households in this cluster tended to be closer to a main road and to have lower time to travel to a market centre than those in the other two clusters. They were in areas with relatively high annual rainfall, were most likely to be surrounded by cropland and least likely to be surrounded by grassland. Households in this cluster tended to be found in areas with the highest human, cattle, sheep, goat, and chicken population densities. They had the smallest cattle herd and goat flock sizes, but with average and median sheep flock sizes broadly equivalent with those in Cluster 2. Household heads in this cluster were most likely to be Meru ethnicity, including 30 (94%) out of all 32 Meru-headed households. Eight (8.5%) out of the 94 Iraqw-headed households, 5 (3.0%) out of the 166 Maasai-, 1 out of 2 Nyiramba-, and 7 (77.8%) out of 9 Nyaturu-headed households were also in this cluster. All of the Hehe- (1) and Chagga- (1) headed households were in this cluster. The proportion of households with heads with at least primary school education was highest in this cluster, as was the proportion of households with a latrine. The majority of households in the cluster reported growing crops, with the proportion of households growing beans, potatoes, and sunflower and reporting selling crops highest in this cluster. Relatively few households in this cluster reported owning donkeys. Ownership of exotic breed cattle and small ruminants was more commonly reported than in the other two clusters. No households in this cluster reported engaging in transhumance. Zero grazing cattle and small stock was common, as was tethering livestock for grazing. Households in this cluster most commonly reported consuming fish in the past three days. This was the smallest cluster (Table 2).

**Fig 5:**
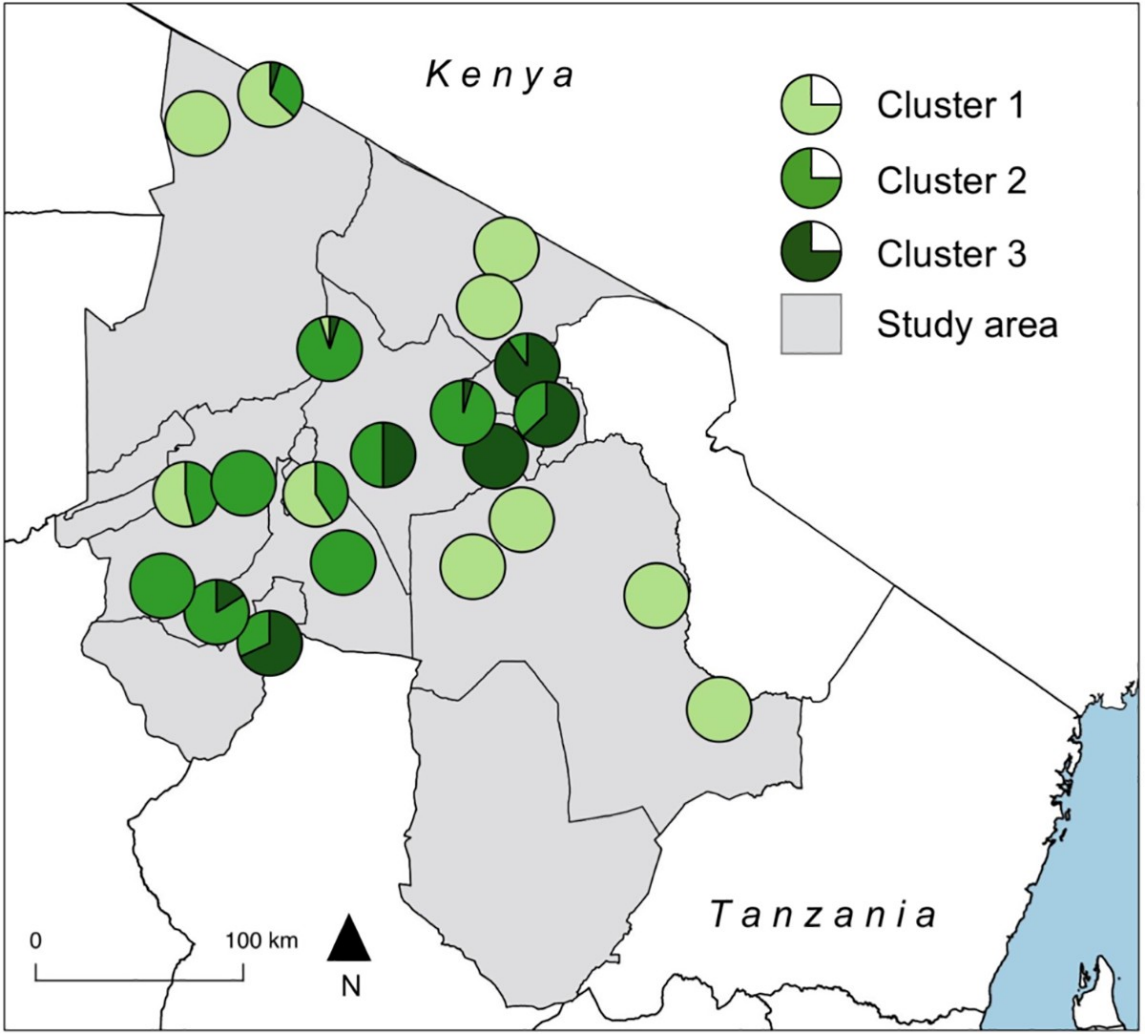
Proportion of households in study villages assigned to each livestock production cluster in northern Tanzania in 2016.

The proportion of households in each village assigned to each of these three clusters is shown in Fig 5. In seven villages, all households were members of Cluster 1; in three villages, all households were members of Cluster 2; and in one village, all households were members of Cluster 3. The remaining 10 study villages comprised a mixture of households from different clusters. Two villages had a mixture of households from all three clusters. When household cluster membership was compared to ‘pastoral’ village membership from the study design stage, 170 (82.9%) households in pastoral villages were in Cluster 1, 34 (16.6%) were in Cluster 2 and 1 (0.5%) was in Cluster 3. When compared to households in ‘mixed’ villages, 1 (0.5%) was in Cluster 1, 143 (71.9%) were in Cluster 2 and 55 (27.6%) were in Cluster 3.

### Variation in household vulnerability between clusters

There were substantial differences in the frequency of reports of adversity-based indicators of household vulnerability between clusters (Table 3). Households in Cluster 1 were most likely to report illness in livestock and people in the previous 12 months, as well as to report livestock losses through mortality. Reports of hunger in the past 12 months were approximately 50% in both Cluster 1 and 2, with the proportion being significantly higher than the average for households in the study area in Cluster 2. Households in Cluster 3 were significantly less likely to report household adversity than average for all indicators under consideration.

## Discussion

Our data analysis identified three clusters of households representing three distinct livestock production systems. The ethnic and production characteristics of these household clusters fit closely into the three traditional typologies of livestock production in northern Tanzania. These are pastoral (cluster 1), agro-pastoral (cluster 2) and smallholder (cluster 3) production systems. Our principal findings are therefore that the traditional livestock production systems that have existed in northern Tanzania for centuries continue to persist, and that the analytical methods used herein can complement more qualitative data categorization methods. While we find no evidence that new typologies of livestock production have emerged, our data suggest changes in production practices within existing systems, including the widespread adoption of crop agriculture among pastoralists. This reflects a trend that has been ongoing in rangeland areas of East Africa for several decades [20,47–49]. Our findings also reveal heterogeneity in a range of indicators of vulnerability between livestock production systems suggesting classification at this level can provide a contextual level for evaluating inequalities in household health and welfare.

There has been a tendency, particularly reflected in livestock and land use policies, for pastoral communities to be viewed as static and resistant to change [50]. In reality, pastoral production systems are characterized by their ability to respond to highly changeable environments [51] and shifts in land use patterns and livelihood diversification in response to both barriers and new opportunities in the pastoralist drylands of northern Tanzanian and southern Kenya are widely documented [11,20,21,48,51–53]. Here, we reveal widespread adoption of non-traditional forms of production within this system, most notably the fact that around three quarters of pastoral households reported practicing crop agriculture over the preceding 12 months, with beans and maize being the most common crop types grown by this group. Approximately 50% of households in this cluster also reported growing crops for more than 10 years. Although crop agriculture has had an often under-appreciated role in East African pastoral livelihoods [22], the frequency with which crop production was reported among pastoral households in this survey indicates that the practice is now very common. Similar findings suggesting recent and widespread adoption of crop agriculture by pastoralists in northern Tanzania have been described by a number of authors [20,21,54]. For example, using recall and longitudinal data collected from communities in Arusha Region, McCabe et al [20] described an increase in the proportion of Maasai-headed households engaging in crop agriculture from less than 5% in the 1940’s to 100% by 1990, with the size of crop holdings also increasing over this period. An important driver for this change is likely to be the need to achieve greater food security as access to grazing lands declines, to increase the security of land tenure, and provide access to additional sources of cash through the sale of crops [20].

Mobility has also often been considered to be a defining characteristic of pastoral households [26]. It is therefore notable that over a quarter of cattle-keeping households in this cluster reported having not used transhumant grazing movements for cattle in the past 12 months, and more than one third of small ruminant keeping households of not using these movements for sheep or goats. It is well known that pastoral communities are undergoing rapid demographic, social, and economic shifts that are likely to influence practices around transhumance [55]. In particular, long-distance livestock movements that have traditionally been a response to variable grass and water availability are widely reported to have become increasingly constrained as a result of competing pressures on traditional grazing lands, including enclosure of previously communal land, conversion to crop lands, and protection for conservation [14,56–59]. It has also been argued that the rise of cultivation within pastoral systems can contribute to reduced mobility and progression towards more sedentary systems in which livestock and crop agriculture are more closely integrated [60]. While our data do not allow us to evaluate changes in herd size, the impacts of restricted grazing and sedenterisation of pastoral communities have been associated with declines in herd sizes in pastoral communities in other settings [61,62].

Our data suggest that crop agriculture now contributes to household livelihoods for the majority of livestock keepers in Arusha and Manyara Regions. While our data analysis clearly demonstrates the ongoing distinctiveness of livestock production in northern Tanzania, production across the two regions included in our study could therefore be considered to be broadly characterised as one of mixed crop and livestock agriculture. Our findings also demonstrate the importance of considering livestock production as part of farming systems in low-income settings more broadly. Historically, this has often not been the case, with support for livestock production reported to form just 1.5% of the total global development assistance for agriculture in 2012 [63]. The presence of under-resourced livestock extension and veterinary services is clearly reflected in our data by the fact that only a quarter of households reported vaccination of animals against any disease, with this being particularly low in agro-pastoral households. Nationally representative household or farm surveys have often focused primarily on crop agriculture and only sparingly on livestock production [64]. Such surveys rarely include questions that are directly relevant to pastoralists, such as practices around nomadism or transhumance. Collection of better livestock data from the range of farming households present in low income settings will contribute to the design and implementation of more effective investment and policies for the agricultural sector [64].

Interventions targeted to pastoralists in the rangeland areas of northern Tanzania are likely to require a “whole farm” focus that includes support for crop agriculture as well as livestock production. Integration of crop and livestock agriculture can contribute to yields and agricultural sustainability [65], but may be limited in crop-producing pastoral households in East Africa [51]. Unsustainable farming practices in combination with the common pastoral imperative to maximize herd sizes may also contribute to further rangeland declines if profits from agriculture are invested in additional livestock [4,51,66]. Strengthening extension services and the promotion of participatory initiatives that can support crop production is likely to be particularly beneficial in communities in which the cultural traditions of agriculture are relatively weak [20]. For example, crop-diversification in semi-arid areas in neighbouring Kenya was positively associated with exposure to agricultural extension officers [67]. Supporting the development of agricultural practices that are resilient to changing climate should also be emphasised. It is notable, for example, that a very small proportion of pastoral households reported growing indigenous crops such as sorghum or millet, which have relatively lower water requirements than introduced maize, and may represent less risky crop choices in dryland areas [68,69]. Household crop diversity was low in all production systems, with maize and beans as the main crops grown. The production of multiple crop types has been linked with lower levels of poverty [70,71] and improved food security [72].

We find that livestock production systems in northern Tanzania are still strongly linked to ethnicity, but that these linkages are not absolute. Livestock-focused interventions and investments are therefore likely to be most effective when based on typologies of production, rather than on ethnicity alone [64]. Almost half of the Barabaig households in our sample were classified as being in the agro-pastoral livestock production system. This relatively small group of traditionally pastoral people is known to have been highly impacted by previous conversion of rangeland areas to commercial crop agriculture [73]. To our knowledge, the long-term impacts of these changes have been infrequently assessed, but the results of our small sample of Barabaig-headed households may point to important changes in livelihood profiles away from pastoralism and towards agro-pastoralism. These changes may also provide a model for a similar process underway in Maasai households. It is also striking that almost 10% of smallholder households were headed by people of Maasai ethnicity. Modern Maasai households may therefore include both pastoral and smallholder farmers, as well as those engaging in a wide range of non-livestock based livelihoods not considered here [74].

A notable finding in our study is the diversity of livestock production systems found within single villages. Previously reported classification systems have tended to classify the dominant livestock production system of large geographic areas [4,34,75–78]. The resulting classification systems have made important contributions to priority-setting, but their regional, continental or global focus has meant that they typically have limited resolution at smaller spatial scales. We show that data-driven, small-area estimation methods that integrate household survey information into farming system classifications can reveal important diversity of livestock production within small areas. Farming system classifications are often used as “recommendation domains”, recognising that members of the same production system are likely to face similar constraints and benefit from similar policy environments. Our findings support the view that broad-scale, landscape-level classifications may be inappropriate for effectively delimiting these domains [79], and that system-wide interventions should recognise the diversity in production that can exist at small spatial scales, including within single villages.

The observed diversity in production systems at the village level may also have implications for household vulnerability. In many systems, be they social, ecological, or economic, increasing diversity tends to be correlated with increased resilience to a range of hazards [80]. It has been argued that the same is true of socio-ecological systems that are centred around livestock production: when systems of reciprocity within a single community are strong, multiple livestock-based livelihood strategies that allow different responses to hazards, such as drought or restrictive policies, can contribute to reducing the vulnerability of the whole community [81]. However, systems of reciprocity within livestock production systems have been substantially eroded in recent times [14,82], and the extent to which these and other types of social capital exist within communities in which a diversity of livestock production typologies exist would be a valuable area for future research. The emergence of greater diversity in production systems at small-spatial scales may also increase the vulnerability of less resilient members of a community. For example, the enclosure of village land through conversion to agricultural land may impact on livestock keepers with larger herd sizes who face resulting grazing restrictions [12–16].

We find important differences in the frequency of reports of a range of indicators of vulnerability between households in the different livestock production systems of northern Tanzania. Our work reveals that the around a half of all households reported hunger over the past 12 months, with the prevalence of reported hunger being high in both pastoral and agro-pastoral production systems. Encouragingly, this prevalence was substantially lower in smallholder households, but such differences reflect profound inequalities in food security between livestock production systems and suggest the need for urgent system-specific interventions. Households in all production systems reported consumption of moderately high levels of animal source food (ASF) in the past three days, but this was lowest in agro-pastoral households. Much of the reported consumption of ASF in agro-pastoral households was through dairy products, with only around a third of households reporting meat consumption compared to over 50% in smallholder households and around 75% of pastoral households. Supplementation of diets with nutrient dense ASF, particularly meat, in areas with low nutritional diversity has been shown to have an important impact on the health and educational attainment of children [83,84]. Dietary diversity has also been strongly linked to food security [85] and to nutritional adequacy [86].

The proportion of households with a government title for land was very low in all systems, and zero in the case of agro-pastoral households. Around one third of households in this group and in the pastoral group also reported land losses in the past 12 months. Such high levels of land insecurity are particularly surprising for agro-pastoralists, which have been described as “settled pastoralists” and could therefore be expected to more commonly have established land rights than pastoralists [87]. Land insecurity is strongly linked to poverty vulnerability and is likely to become an increasing issue with population growth in the region [56]. Efforts in pastoral communities have been made by local non-governmental organisations to facilitate the securing of land titles and land rights, but with mixed effect [32]. Our data demonstrate that there is a clear need to improve land security in northern Tanzania, with this need greatest in both pastoral and agro-pastoral livestock production systems.

We find no differences between production systems in the proportion of households with a male head or the proportion that treat water before drinking, which was low in all settings. However, household-level latrine ownership was significantly lower than average in pastoral households. Some of the differences between production system in latrine access are likely to be explained by cultural differences influenced by greater mobility and lower human population densities in pastoral communities that may result in higher levels of open defaecation. The absence of a latrine has been linked to household-level infectious disease risk [88] as well as to reduced childhood growth and development [89]. Households in the pastoral livestock production system can therefore be expected to benefit from exposure to locally appropriate models for pit latrines, with culturally appropriate engagement to encourage their use. Our data also indicate that pastoral households are most likely to report illness in people and in livestock, and mortality in livestock. These results need to be interpreted with some caution as they are not adjusted for household or livestock numbers, the latter of which was largest in pastoral households. However, they may suggest a higher prevalence of poor human and animal health in these communities. Higher prevalence of exposure to a range of infectious diseases is commonly described for livestock reared in pastoral settings when compared to other settings [39,40,90], with larger herd sizes, high levels of mobility, and greater between herd contacts at shared grazing and watering resources likely explanations for increased risk. Human health differences between ethnic groups in northern Tanzania have been previously described, with children in Maasai-headed households three times more likely to be stunted than Meru children, as well as being more likely to report diarrhea, fever, and respiratory illness [91]. Further work is needed to understand the importance of livestock production system as a contextual level in shaping risk for both communicable and non-communicable human disease in northern Tanzania.

The smallholder production system had the lowest proportion of households reporting hunger, illness in people or livestock, livestock or crop losses or land losses. Smallholders also tended to report the widest diversity of food consumption and had the highest level of household head education and latrine ownership. While we did not collect detailed data on household inputs as part of this study, smallholder systems in northern Tanzania have historically represented very high levels of agricultural intensification [23]. The apparent resilience of households within this system may support links between agricultural intensification and prosperity [92], which is promoted as route to poverty reduction for rural households [93]. This group was found in peri-urban areas and are therefore also likely to benefit from greater access to markets, extension and other services, as well as non-agricultural sources of income that were not recorded here. Livestock producers described as “smallholders” are often a focus for development interventions in the livestock sector in sub-Saharan Africa [94]. However, based on the indicators of vulnerability explored in this study, and given scarce resources, livestock-keeping households in agro-pastoral and pastoral settings in northern Tanzania appear to be in considerably greater need of support for poverty alleviation. It is important to note, however, that the production systems we describe here represent those in two regions of northern Tanzania, and ‘smallholders,’ ‘agro-pastoralists’ and ‘pastoralists’ may have different characteristics and different levels of vulnerability in other parts of the country and internationally. Future studies that use a similar approach to that described here to classify livestock production systems in other geographic areas would provide further understanding of the diversity of livestock production that exists in Tanzania and in sub-Saharan Africa more broadly.

A number of limitations should be considered when interpreting our findings. Households were selected from a limited number of villages, and villages in urban areas were excluded from the sampling procedure. Livestock production commonly occurs in urban areas of Tanzania where it tends to be characterised by small scale, intensive zero-grazing production of cattle and small ruminants that could be expected to fall into the smallholder classification. The proportion of households that were categorised as smallholder was smaller than those in the other systems. With a larger sample, greater diversity within the smallholder system may have emerged, potentially including the classification of distinct typologies. In particular, while intensive livestock production methods based around zero grazing were very commonly reported in the smallholder system, relatively few households reported ownership of high yielding, European-breed dairy cattle or the sale of milk. Hence, greater sampling in smallholder settings, including in areas classified as ‘urban’ may have revealed a distinct typology involving high yielding European breed cattle or their crosses kept exclusively for commercial purposes.

A related limitation is that all livestock keeping households included in this study were those who attended a central point sampling event, which captured only a proportion of livestock keeping households in most villages. There is therefore the potential for selection bias to have been introduced if characteristics of households made them more or less likely to attend with their animals. This may be particularly important for those households in the smallholder sector, where zero-grazing of animals may have influenced attendance. In particular, households with high value, European dairy breed cattle may have been reluctant to attend the sampling event, further limiting our ability to identify an emerging typology of commercial dairy production in the study area. Self-selection of study participants may also have impacted on our ability to estimate the proportion of households engaging in transhumance, since members of such households may have been away from the sub-village at the time of sampling. However, transhumant households tend keep some animals at home in order to meet the nutritional needs of remaining household residents as the main herd or flock is moved for grazing. Such households could therefore still be expected to attend the sampling event.

Our ability to identify livestock keepers in the commercial sector was further limited by the fact that our survey included relatively little information on market integration beyond the sale of crops and milk and travel time to market centres. Further information would be required to distinguish between livestock producers who are principally subsistence-orientated from those who are more market-orientated. Each group is likely to face very different constraints and benefit from different policy environments and interventions. In the case of the former, improvements in livestock health and productivity could be expected to improve household health and productivity while in the case of the latter, investments may also have wider economic impacts, for example through employment generation [64]. Despite these limitations, our data indicate only a minority of livestock keepers were engaged in crop sales and a smaller minority in milk sales, with engagement particularly low in pastoral and agro-pastoral households. Enhanced market integration can improve household food security and is considered to be key to poverty alleviation for small- and medium scale farmers [93].

A further limitation of our data and the analysis presented here is that we have not included information on livelihood diversification or on household migration. Both represent potentially important alternative livelihood strategies beyond household crop and livestock production for the rural poor. In the absence of information on these off-farm dimensions, the focus of our analysis should be considered to be on farming systems rather than on rural livelihood systems more broadly. Moreover, while we included a wide range of household-level characteristics, and the resulting clusters reflect expected and sensible groupings of livestock-keeping households with these characteristics in this region of Tanzania, dimension reduction and hierarchical clustering approaches are sensitive to input data. We therefore cannot rule out that the inclusion of a wider (or smaller) range of household level variables than were available to us may have resulted in a different number of clusters, or clusters with different general characteristics. A valuable area for future work would be to explore how the clustering we identified depends on key variables, such as ethnicity, livestock numbers, or local environment. Identification of a more parsimonious set of indicators of livestock production that can be integrated into surveys of farming households in order to enable standardised classification into production system using the data driven methods described here would be particularly valuable.

Finally, our survey data are cross-sectional and were not designed to measure change in household-level characteristics over time. However, studies such as our own can provide an important baseline to enable future studies to measure such change.

## Conclusion

System-level interventions can improve the food security and reduce the poverty of rural livestock keepers. Here, we show that three livestock production systems exist in northern Tanzania which are broadly equivalent to traditional typologies of livestock production that are known to have existed in the area for centuries. These production systems are distinct in a range of characteristics, including local climate and land use practices, human and animal population density, distance to markets and services, household demographics and resources, livestock type and numbers, livestock management, and food consumption practices. The majority of livestock keeping households grow crops, with the area as a whole constituting a diversity of mixed crop and livestock production practices. We also show substantial variation in several indicators of vulnerability between production systems, reflecting important inequalities in human health and well-being at the livestock production system level. On- and off-farm interventions are required to reduce the vulnerability of livestock keepers in northern Tanzania and should recognise the continuing distinctiveness of pastoral, agro-pastoral and smallholder production systems in the region, but also the widespread adoption of crop agriculture by livestock keepers. Importantly, those developing such interventions should recognise that a diversity of production systems can exist at small spatial scales, including within a single village.

## Supporting information

S1 File Supplementary materials. (PDF)
