# Supplementary material for: Classification and characterisation of livestock production systems in northern Tanzania
Source: PLoS One. 2020 Dec 30;15(12):e0229478. doi: 10.1371/journal.pone.0229478 (PMC7773236; doi:10.1371/journal.pone.0229478)
Supplement: S1 File — (PDF) [file pone.0229478.s001.pdf]

Classification and characterisation of livestock production systems in northern Tanzania

de Glanville W.A. et al.

Supplementary materials

The following questions asked during the household survey were used to derive data for variables in Domains 2, 3, 4, 5, 6, 7 and 8 in Table 1 (in the main text). The variable name (from Table 1) the question contributes to is given in bold.

1. What is the head of household's gender [*Jinsia ya mkuu wa kaya*]? (**Sex of household head**)

1. What is your [head of household] tribe [Kabila lako ni lipi]? (*Options: Arusha; Maasai; Barabaig; Chagga; Iraqw; Pare; Sambaa; Other (specify)*) (**Maasai, Arusha, Meru, Iraqw, Barabaig, Nyaturu ethnicity**)

2. What is the highest level of education of the head of the household [Kiwango cha juu cha elimu cha mkuu wa kaya]? (*No formal education; Some primary school; Completed primary school; Some secondary school; Completed secondary school; Post-secondary qualifications; Some university; University completed; Post graduate*) (**Household head completed primary school**)

3. How frequently do you treat water before drinking it [Huwa unatibu maji ya kunywa mara nyingi kabla ya kunywa]? (*Always (daily); Sometimes (a few times a week); Rarely (a few times a month); Never*) (**Treat drinking water**)

4. What type of toilet system do members of this household use [Ni aina gani ya mfumo wa choo ambao unatumika kawaida na kaya yako]? (*Flush or pour toilet with septic tank, including squat toilet; Flush or pour toilet connected to sewer pipe; Pit latrine with covering slab; Pit latrine without covering slab; Ventilated improved pit latrine (VIP); Bucket or plastic bags; No facilities (use field/bush); Other*) (**Has latrine**)

5. Do members of this household own any of the following items [Je, wakazi wa kaya hii wanamiliki chochote kati ya vitu vifuatavyo]? (*Ox plough; Ox cart; Bicycle; Motorbike; Car; Tractor; Mobile phone; Radio; Television; Sofa; Refrigerator; None of the above*) (**Own plough**)

6. Do members of this household own land [Je, wanakaya wa kaya hii wanamliki ardhi]? (*Yes; No*) **(Government title for land)**

7. Do members of the household have a title for this land [Je, wanakaya wa kaya hii wana hati miliki ya hii ardhi]? (*Yes; No*) **(Government title for land)**

8. Where is the land title from [Je, hati miliki ya hii ardhi imetoka wapi]? (*Local (traditional); Government; Other*) **(Government title for land)**

9. Were any crops grown by members of this household during the last 12 months [Kuna mazao yeyote yalioteshwa na watu wa kaya hili katika miezi 12 iliyopita]? (*Yes; No*) (**Grow no crops**)

10. Which crops did members of the household grow in the past 12 months [Kwa miezi 12 iliyopita ni mazao gani yalioteshwa na watu wa kaya hili]? (*Avocado; Banana/Plantain; Beans; Cabbage; Cassava; Coffee; Cotton; Kale; Lettuce; Maize; Millet; Potato; Rice; Sesame; Sorghum; Spinach; Sugar cane; Sunflower; Sweet potato; Tomato; Wheat; Other*) (**Grow crop types**)

11. How many years have members of this household been growing crops [Ni kwa miaka mingapi watu wana kaya hii wameotesha mazao]? (**Growing crops > 10 years**)

12. Did this household sell any crops in the last 12 months [Je, umeuza mazao yoyote katika miezi 12 iliyopita]? (*Yes; No*) (**Sell crops**)

13. How many months do staple crops (e.g. beans, maize, rice, bananas) support your family in an average year [Ni kwa miezi mingapi hayo mazao ya chakula (maharage, mahindi, mchele, ndizi) yanaweza kulisha familia yako kwa wastani katika mwaka]? (**Supplies of staple crops last 6 months or more**)

14. Are any cattle currently kept at this compound [Je, kuna ng'ombe wowote wanafugwa kwenye boma hili kwa sasa]? (*Yes; No*) (**Number of cattle**)

15. What is the total number of cattle that are managed together at this compound [Kuna jumla ya ng'ombe wangapi wanaotunzwa pamoja katika boma hili]? (**Number of cattle**)

16. How many of these cattle are exotic breeds [Wangapi kati ya hawa ni ng'ombe wa kisasa]? (**Own exotic breed cattle**)

17. Are any goats currently kept at this compound [Je, kuna mbuzi wowote wanafugwa kwenye boma hili kwa sasa]? (*Yes; No*) (**Number goats**)

18. What is the total number of goats that are managed together at this compound [Kuna jumla ya mbuzi wangapi wanaotunzwa pamoja katika boma hili]? (**Number goats**)

19. How many of these goats are exotic breeds [Wangapi kati ya hawa ni mbuzi wa kisasa]? (**Own exotic breed small ruminants**)

20. Are any sheep currently kept at this compound [Je, kuna kondoo wowote wanafugwa kwenye boma hili kwa sasa]? (*Yes; No*) (**Number sheep**)

21. What is the total number of sheep that are managed together at this compound [Kuna jumla ya kondoo wangapi wanaotunzwa pamoja katika boma hili]? (**Number sheep**)

22. How many of these sheep are exotic breeds [Wangapi kati ya hawa ni kondoo wa kisasa]? (**Own exotic breed small ruminants**)

23. [Do you own] Donkeys [Punda]? (*Yes; No*) (**Own donkeys**)

25. [Do you own] Pigs [Nguruwe]? (*Yes; No*) (**Own pigs**)

27. [Do you own] Chickens [Kuku]? (*Yes; No*) (**Own chickens**)

29. In the past month have you sold milk from animals in this household [Katika mwezi uliopita umeuza maziwa kutoka kwa wanyama katika kaya hii]? (*Yes; No*) (**Sell milk**)

30. Have any animals in your herd been vaccinated in the past 24 months [Je, kuna wanyama katika kundi wamepata chanjo katika kipindi cha miezi 24 iliyopita]? (*Yes; No*) (**Vaccinate against any disease**)

31. How are cattle in this compound grazed during the wet and dry season [Unawalishaje ng'ombe wa boma hili kipindi cha mvua na kiangazi]? (*Free grazing (no herdsperson); Herded; Tethered; Zero grazed*) (**Zero graze cattle, tether cattle**)

32. How are goats in this compound grazed during the wet and dry season [Unawalishaje mbuzi wa boma hili kipindi cha mvua na kiangazi]? (*Free grazing (no herdsperson); Herded; Tethered; Zero grazed*) (**Zero graze goats, tether small stock**)

33. How are sheep in this compound grazed during the wet and dry season [Unawalishaje kondoo wa boma hili kipindi cha mvua na kiangazi]? (*Free grazing (no herdsperson); Herded; Tethered; Zero grazed*) (**Zero graze sheep, tether small stock**)

34. Which of the following best describes the way you manage the herding of animals in this compound [Ipi kati yafuatayo inaeleza vizuri jinsi unavyotunza kundi la wanyama katika boma hili]? (*Cattle, sheep and goats together; Cattle separately, sheep and goats together; Cattle with goats, sheep separately; Cattle with sheep, goats separately; All species separately; Other*) (**Graze cattle with small stock**)

35. Are cattle from this compound regularly taken to seasonal camps for grazing [Je, ng'ombe wa boma hili wanapelekwa kwenye maboma ya muda kwa ajili ya malisho (ronjo)]? (*Yes; No*) (**Cattle transhumance**)

36. Are goats from this compound regularly taken to seasonal camps for grazing [Je, mbuzi wa boma hili wanapelekwa kwenye maboma ya muda kwa ajili ya malisho (ronjo)]? (*Yes; No*) (**Small stock transhumance**)

37. Are sheep from this compound regularly taken to seasonal camps for grazing [Je, kondoo wa boma hili wanapelekwa kwenye maboma ya muda kwa ajili ya malisho (ronjo)]? (*Yes; No*) (**Small stock transhumance**)

38. In the past 4 days have you or anyone else in the household eaten the following foods [Katika siku 4 zilizopita wewe au mtu yeyote katika kaya yako alikula vyakula vifuatavyo]? (*Maize, ugali, chapati, rice, bread, plantains, noodles, millet, or any other food made from rice, wheat, millet or sorghum; Potatoes, yams, cassava, or any other foods made from roots or tubers; Vegetables or fruits; Chicken, duck or other birds (including liver, kidney, heart, or other organ meats from such animals); Beef, pork, lamb, goat, rabbit, wild game or liver, kidney, heart or other organ meats from these animals; Eggs; Fresh or dried fish or shellfish; Foods made from beans, peas, lentils, nuts; Cheese, yogurt, milk or other milk products; Foods made with oil, fat, or butter; Blood from livestock, either fresh or processed; None of the above*) (**All consumption variables**)

39. Did your household experience any of the following situations during the past year [Je, kaya yako ilipata yeyote kati ya hali zifuatazo katika mwaka uliopita]? (*Hunger; Long illness (people); Death; Loss of job; Theft; Livestock illness; Damage/loss of your home; Shortage of labour for herding livestock; Loss of crops; Loss of livestock (death, predation, theft, etc.); Other*) (**All vulnerability variables**)

Table S1 gives the source of the data used for variables in Domain 1 shown in Table 1 (in the main text).

Table S1. Source and description of continuous variables used to represent household level environmental characteristics in northern Tanzania.

Variable	Source	Description
Average annual vegetation cover	https://lpdaac.usgs.gov/products/mod13q1v006/	Annual yearly average enhanced vegetation index (EVI)
Distance to main road	http://www.fao.org/geonetwork/srv/en/main.home	Trunk roads extracted. Euclidean distance between household and nearest trunk road calculated in kilometres using the Distance Matrix tool in QGIS
Time to travel to market centre	https://harvestchoice.org/labs/travel-time-major-market-cities	Travel time to a town or city of greater than 20,000 people in hours
Total annual precipitation	www.worldclim.org/bioclim	Annual precipitation in mm (BIO12)
Average annual temperature	www.worldclim.org/bioclim	Average annual mean temperature in °C (BIO1)
Maximum slope	https://lta.cr.usgs.gov/srtmgl3.html	Slope in degrees calculated from SRTM data using the terrain analysis tool in QGIS. Maximum estimated from area with 1 km radius around each household
Local crop land cover	https://landsat.gsfc.nasa.gov/data	Proportion of an area with 1 km radius around each household classified by Landsat as cropland
Local grassland cover	https://landsat.gsfc.nasa.gov/data	Proportion of an area with 1 km radius around each household classified by Landsat as grassland
Local forest cover	https://landsat.gsfc.nasa.gov/data	Proportion of an area with 1 km radius around each household classified by Landsat as forest
Local human population density	www.worldpop.org.uk/	Population density data extracted for Tanzania in 2016 in Km^2
Area of village	https://www.nbs.go.tz	Area in decimal degrees calculated for shapefile of village boundaries
Local cattle density (km^2)	www.fao.org/ag/againfo/resources/en/glw/GLW_dens.html	Data extracted from global cattle distribution in 2006 (Global livestock of the world (GLW) 2)
Local sheep density (km^2)	www.fao.org/ag/againfo/resources/en/glw/GLW_dens.html	Data extracted from global sheep distribution in 2006 (GLW 2)
Local goat density (km^2)	www.fao.org/ag/againfo/resources/en/glw/GLW_dens.html	Data extracted from global goat distribution in 2006 (GLW 2)
Local chicken density (km^2)	www.fao.org/ag/againfo/resources/en/glw/GLW_dens.html	Data extracted from global chicken distribution in 2006 (GLW 2)
Local pig density (km^2)	www.fao.org/ag/againfo/resources/en/glw/GLW_dens.html	Data extracted from global pig distribution in 2006 (GLW 2)

Figure S1 shows the scree plot of eigenvalues associated with each factor. A natural break is observed between Factor number 5 and 6.

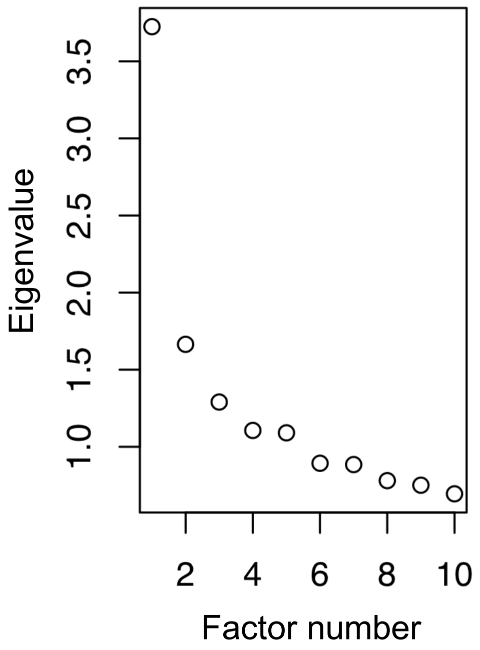

Figure S1. Scree plot of eigenvalues associated with each factor derived from the MFA applied to livestock keeping households in northern Tanzania

Table S2 provides the scores and relative contributions to the first and second factor for each categorical variable used in the MFA procedure. Table S3 provides the scores and relative contributions to the first and second factor for each continuous variable used in the MFA procedure.

Table S2. Scores and relative contribution assigned to each categorical variable for the first and second dimensions in the MFA applied to livestock keeping households in northern Tanzania.

Variable (*domain*)	Dim. 1 scores	Dim. 2 scores	Contribution to Dim. 1 (%)	Contribution to Dim. 2 (%)
Household				
Household head female	0.79	0.71	0.16	0.64
Household head male	-0.07	-0.06	0.01	0.05
Household head non-Maasai	1.28	-0.21	3.13	0.41
Household head Maasai	-1.83	0.29	4.48	0.58
Household head non-Arusha	-0.08	0.08	0.02	0.07
Household head Arusha	0.33	-0.30	0.07	0.29
Household head non-Meru	-0.20	-0.17	0.12	0.42
Household head Meru	2.35	1.93	1.43	4.83
Household head non-Iraqw	-0.55	0.32	0.76	1.30
Household head Iraqw	1.82	-1.06	2.52	4.30
Household head non-Barabaig	0.02	0.01	0.00	0.00
Household head Barabaig	-0.80	-0.54	0.04	0.10
Household head non-Nyaturu	-0.05	-0.03	0.01	0.02
Household head Nyaturu	2.24	1.46	0.37	0.77
No primary school	-0.90	-0.06	1.35	0.03
Primary school completed	0.93	0.06	1.40	0.03
No government title for land	-0.03	-0.07	0.00	0.09
Government title for land	0.80	1.79	0.08	2.07
No latrine present	-1.70	0.00	3.06	0.00
Latrine present	0.82	0.00	1.47	0.00
Do not treat water	-0.03	0.03	0.00	0.01
Treat water	0.08	-0.07	0.01	0.02
Crops				
Grow crops	0.32	-0.10	0.20	0.10
Grow no crops	-2.19	0.69	1.34	0.67
Not growing crops > 10 years	-1.53	0.46	1.42	0.63
Growing crops > 10 years	0.60	-0.18	0.56	0.25
No beans	-0.76	0.05	0.49	0.01
Beans	0.49	-0.03	0.32	0.01
No maize	-1.87	0.47	1.60	0.51
Maize	0.50	-0.13	0.43	0.14
No millet	-0.08	0.06	0.01	0.04
Millet	0.94	-0.72	0.15	0.43
No potato	-0.10	0.01	0.02	0.00
Potato	2.05	-0.22	0.43	0.03

No sesame	-0.03	0.03	0.00	0.01
Sesame	1.03	-0.81	0.07	0.23
No sorghum	-0.04	0.04	0.00	0.02
Sorghum	1.16	-1.26	0.09	0.51
No sunflower	-0.17	0.04	0.06	0.02
Sunflower	1.67	-0.43	0.57	0.19
No wheat	-0.02	0.02	0.00	0.00
Wheat	0.77	-0.63	0.04	0.13
No onions	0.15	-0.03	0.05	0.01
Onions	-2.44	0.43	0.77	0.12
No cowpeas	-0.07	0.06	0.01	0.04
Cowpeas	1.15	-0.97	0.17	0.61
Staple crops do not last 6 months	-1.04	0.29	0.95	0.37
Staple crops last 6 months	0.71	-0.20	0.65	0.25
No plough	-0.51	-0.03	0.33	0.00
Plough	0.71	0.04	0.46	0.01
No crop sales	-0.32	0.07	0.14	0.03
Crop sales	0.55	-0.12	0.24	0.06
Livestock type				
No exotic cattle	0.00	-0.11	0.00	0.33
Exotic cattle	0.14	3.97	0.00	11.68
No exotic goats	-0.01	-0.08	0.00	0.17
Exotic goats	0.50	3.46	0.03	7.26
No pigs	-0.28	0.24	0.36	1.41
Pigs	1.91	-1.68	2.52	9.73
No donkeys	1.28	0.16	3.79	0.29
Donkeys	-0.94	-0.12	2.78	0.21
No chickens	-1.15	0.48	1.02	0.90
Chickens	0.19	-0.08	0.17	0.15
Livestock management				
No cattle transhumance	1.13	-0.09	2.13	0.07
Cattle transhumance	-1.78	0.14	3.35	0.11
No small stock transhumance	0.81	-0.10	1.28	0.09
Small stock transhumance	-1.99	0.24	3.14	0.23
Cattle not grazed with small stock	-0.40	0.16	0.33	0.25
Cattle grazed with small stock	1.21	-0.47	0.99	0.75
No cattle zero grazing	-0.29	-0.26	0.20	0.86
Cattle zero grazing	2.53	2.35	1.76	7.62
No small stock zero grazing	-0.26	-0.24	0.16	0.71
Small stock zero grazing	2.55	2.37	1.62	6.99
No cattle tethering	-0.11	-0.04	0.03	0.02
Cattle tethering	2.30	0.79	0.68	0.40

No small stock tethering	-0.11	-0.03	0.03	0.01
Small stock tethering	2.14	0.52	0.62	0.18
No vaccination	0.34	-0.10	0.24	0.11
Vaccination against any disease	-1.13	0.34	0.80	0.35
No milk sales	-0.04	-0.17	0.00	0.35
Sell milk	0.22	0.98	0.02	1.98
Food consumption				
No meat consumption	0.59	-0.29	0.48	0.60
Meat consumption	-0.50	0.25	0.41	0.51
No dairy consumption	0.04	0.34	0.00	0.49
Dairy consumption	-0.02	-0.13	0.00	0.20
No blood consumption	0.09	-0.04	0.02	0.03
Blood consumption	-1.22	0.59	0.30	0.36
No vegetable consumption	-1.25	-0.07	1.45	0.02
Vegetable consumption	0.54	0.03	0.63	0.01
No legume consumption	-0.47	0.05	0.22	0.01
Legume consumption	0.24	-0.02	0.11	0.01
No fats consumption	-0.03	0.15	0.00	0.16
Fats consumption	0.02	-0.12	0.00	0.13
No fish consumption	-0.23	-0.13	0.14	0.22
Fish consumption	1.82	1.01	1.12	1.73
No poultry consumption	-0.15	-0.03	0.06	0.01
Poultry consumption	0.86	0.14	0.34	0.05
No roots consumption	-0.30	-0.05	0.20	0.03
Roots consumption	0.75	0.14	0.49	0.08
No eggs consumption	-0.25	0.00	0.15	0.00
Eggs consumption	0.90	-0.01	0.53	0.00
No animal source foods	0.07	-0.11	0.00	0.02
Animal source food consumption	-0.01	0.01	0.00	0.00
Vulnerability				
No hunger reported	0.30	0.28	0.14	0.64
Hunger reported	-0.36	-0.34	0.17	0.78
No illness reported	0.79	0.55	0.71	1.71
Illness reported	-0.51	-0.35	0.46	1.10
No illness in livestock reported	0.69	0.41	0.63	1.13
Illness in livestock reported	-0.57	-0.34	0.53	0.93
No crop losses reported	0.03	0.26	0.00	0.66
Crop losses reported	-0.06	-0.49	0.00	1.23
No livestock losses reported	0.62	0.25	0.66	0.53
Livestock losses reported	-0.87	-0.35	0.92	0.74
No land loss reported	0.12	0.23	0.03	0.54
Land loss reported	-0.32	-0.59	0.08	1.42

Table S3. Scores and relative contribution assigned to each continuous variable for the first and second dimensions in the MFA applied to livestock keeping households in northern Tanzania.

Variable (*domain*)	Dim. 1 scores	Dim. 2 scores	Contribution to Dim. 1 (%)	Contribution to Dim. 2 (%)
Environment				
Average annual vegetation cover	0.6	-0.29	1.21	0.71
Distance to main road (km)	-0.1	-0.35	0.04	1.08
Travel time to market centre (hours)	-0.3	-0.47	0.3	1.87
Total annual precipitation (mm)	0.6	0.11	1.53	0.10
Average annual temperature (°C)	-0.4	0.14	0.74	0.17
Maximum slope (degrees)	0.5	-0.14	0.79	0.17
Local cropland cover (%)	0.6	0.11	1.59	0.10
Local grassland cover (%)	-0.6	-0.11	1.57	0.10
Local forest cover (%)	0.1	-0.07	0.08	0.04
Local human population density (km^2)	0.8	0.03	2.23	0.01
Area of village (decimal degrees^2)	-0.8	-0.14	2.46	0.17
Local cattle population density (km^2)	0.7	0.30	1.87	0.76
Local sheep population density (km^2)	0.6	0.32	1.44	0.90
Local goat population density (km^2)	0.5	0.30	1.06	0.79
Local chicken population density (km^2)	0.7	0.28	1.82	0.66
Local pig population density (km^2)	0.6	-0.02	1.22	0.00
Livestock				
Number of cattle	-0.5	0.11	3.02	0.30
Number of goats	-0.6	0.17	3.82	0.72
Number of sheep	-0.4	0.16	2.12	0.66
